# A study on the cervical spondylotic myelopathy treated by anterior cervical diskectomy and fusion in accordance with Modic changes with a 2-year minimum follow-up

**DOI:** 10.1186/s13018-014-0146-8

**Published:** 2015-01-28

**Authors:** Jia Li, Yongqian Li, Jingchao Wei, Yong Shen

**Affiliations:** Department of Orthopaedic Surgery, The Third Hospital of Hebei Medical University, The Key Laboratory of Orthopedic Biomechanics of Hebei Province, 139 Ziqiang Road, Shijiazhuang, 050051 China; Department of Orthopedic Surgery, Hebei General Hospital, 348 Heping Road, Shijiazhuang, 050000 China

## Abstract

**Background:**

The aim of this research is to analyze the influence of Modic types on the clinical results of cervical spondylotic myelopathy treated by anterior cervical diskectomy and fusion.

**Methods:**

A total of 106 patients with a mean age of 55.8 ± 6.5 years were included in this study. Patients with Modic changes were retrospectively reviewed. In this study, 23 patients were classified as Modic-1, 39 patients were classified as Modic-2, and 44 patients were classified as Modic-0. Clinical evaluations were performed preoperatively and repeated at 3, 6, 12, and 24 months after operation.

**Results:**

In this study, all patients were followed up for a mean period of 30.2 months (range, from 24 to 36 months). Significant clinical improvement (*P* < 0.05) was observed in Japanese Orthopaedic Association (JOA) score and axial symptoms between the preoperative evaluation and the final follow-up. Comparing the result of mean JOA score after anterior cervical diskectomy and fusion (ACDF) in the Modic-1 group and other groups, statistically significant differences could be found at 12 months after surgery (*P* < 0.05). Comparing the outcome visual analog scale (VAS) of axial symptoms among different groups after ACDF, patients with Modic-1 changes showed significantly lower VAS of axial symptoms postoperatively (*P* < 0.05).

**Conclusion:**

After anterior cervical diskectomy and fusion, both Modic-1 and Modic-2 groups showed excellent clinical outcomes over a 2-year follow-up. Better clinical results were achieved in patients with Modic-1 changes compared to the group of patients with Modic-2 and Modic-0 changes on magnetic resonance images.

## Introduction

Cervical spondylotic myelopathy (CSM) is caused by spinal cord compression which is a common consequence of degenerative disk disease. Anterior cervical diskectomy and fusion (ACDF) is the most commonly used surgical treatment for degenerative disk disease of the cervical spine [1-3]. After the surgical intervention, some patients were not satisfied with functional recovery, especially the relief of the axial symptoms [4]. However, the key factor that influenced the functional recovery was still unknown. Many factors might affect the postoperative result, such as gender, age, and duration of compression.

In 1988, Modic et al. [5] characterized signal abnormalities of the vertebral endplates on magnetic resonance images (MRI). The Modic changes were classified into types 1, 2, and 3. Modic changes, regardless of type, have been shown to be associated with degenerative changes of the intervertebral disk and chronic low back pain [6-8]. The previous studies focused on the relationship between lumbar disk degeneration and Modic change development. Although the precise clinical relevance of Modic changes is a controversy, there have been many studies to explore the relation between Modic change and chronic low back pain. They also have attempted to correlate Modic changes with clinical outcome following lumbar surgery [7-10].

Modic changes are also observed in the cervical spine. Peterson et al. [11] had reported that Modic-1 changes were common in the cervical spine, which is not similar to the lumbar spine. However, to our knowledge, there has been no literature which is specifically to study the influence of Modic change type on outcomes following ACDF. The aim of this study is to evaluate the influence of Modic change type on the clinical outcome after ACDF.

## Material and methods

From 2005 to 2010, in our institution, 106 patients who underwent one-level ACDF between C4 and C7 for degenerative disk disease were chosen. After informed consent and approval by the institutional review board, a total of 57 men and 49 women whose mean age was 55.8 ± 6.5 years (40–65 years) were included in this study.

One of the inclusion criteria included the following: patients with chronic axial symptoms [12] resulting from single-level cervical disk degeneration, which is confirmed by cervical MRI and nonresponsive to appropriate nonsurgical treatment for at least 6 months. Patients with cervical axial symptoms nonrelated to disk degeneration, multilevel disk degeneration, prominent radicular pain, associated spine deformities (scoliosis and/or spondylolisthesis), tumor spinal pathologies, spinal infections, and acute spinal trauma were excluded. Smokers were not excluded. This study was approved by the Institutional Review Board of the Third Hospital of Hebei Medical University. Signed informed consent was obtained from each patient. The clinical investigations were conducted following the principles expressed in the Declaration of Helsinki. All of the patients were informed that they were going to be in this study, and those who did not wish to participate in this study were not enrolled.

### Imaging assessment

**Figure 1:**
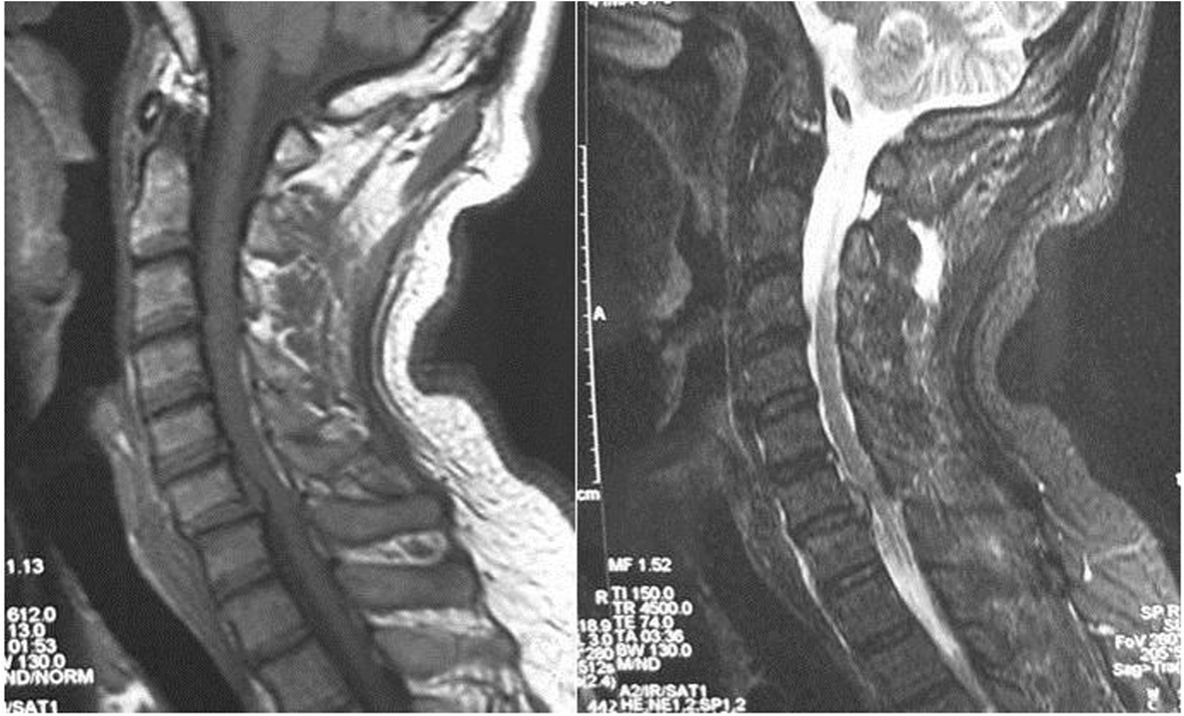
**T1-weighted (left) and T2-weighted (right) images demonstrate C6-C7 with Modic-1 changes on MRI.**

**Figure 2:**
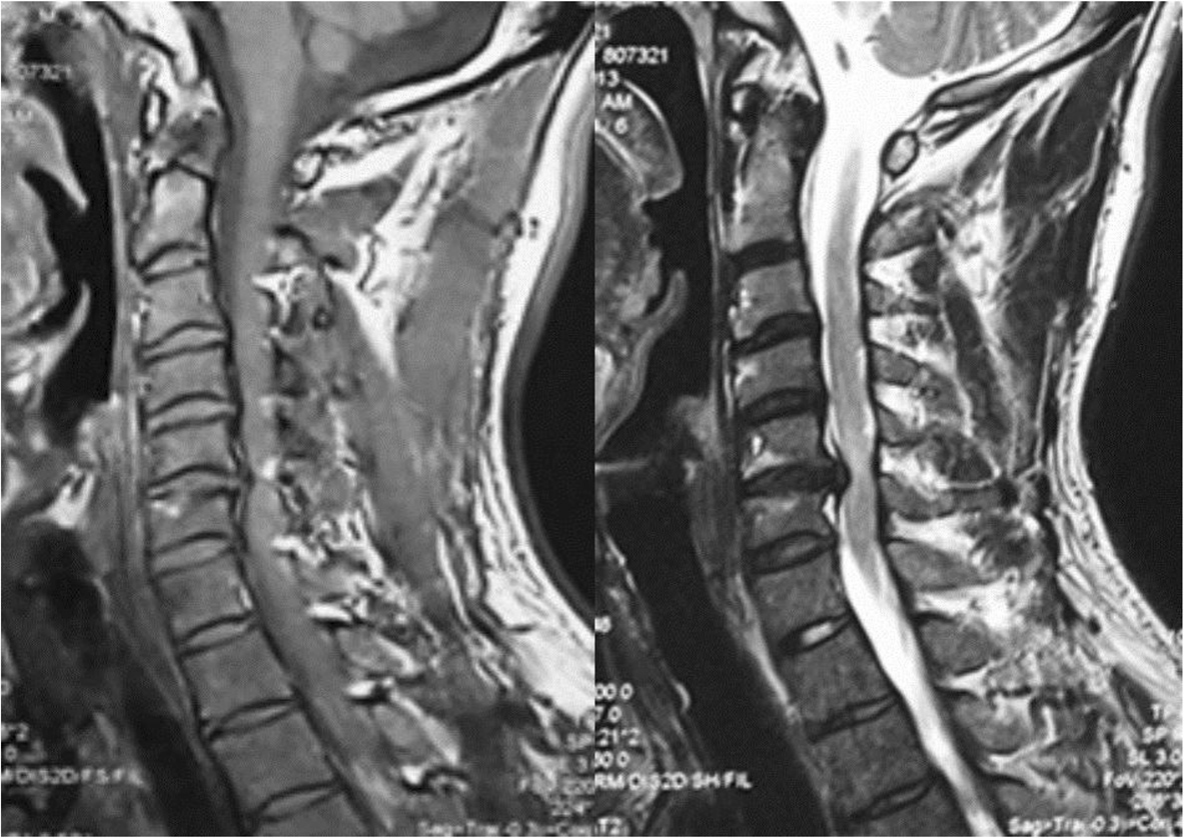
**T1-weighted (left) and T2-weighted (right) images demonstrate C5-C6 with Modic-2 changes on MRI.**

**Figure 3:**
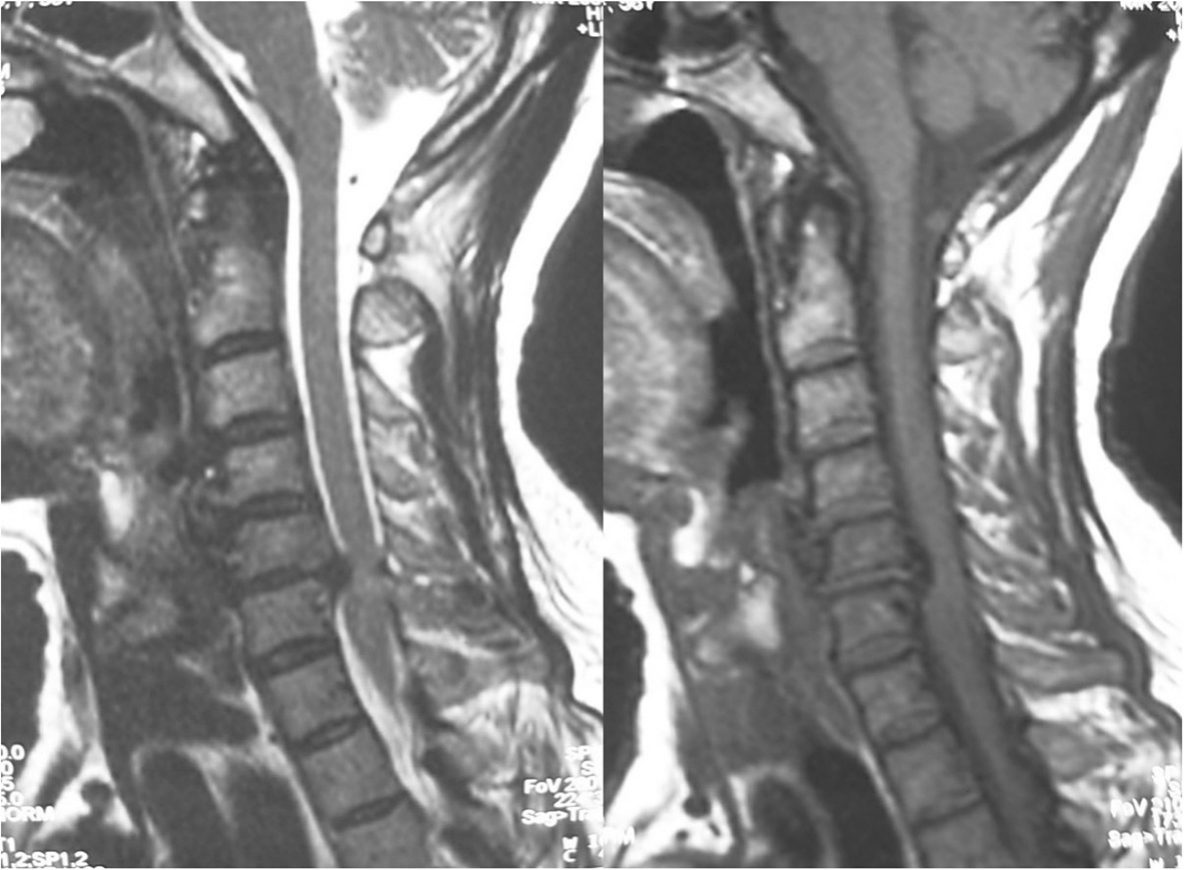
**T1-weighted (left) and T2-weighted (right) images demonstrate disk degeneration without Modic endplate change (Modic-0).**

Before operation, all patients received high-resolution MRI with a 1.5-T (SIEMENS MAGNETOM Symphony, Germany) imager. T1-weighted images (T1WIs) and T2-weighted images (T2WIs) of sagittal views of the cervical cord were obtained by a spin echo sequence system for T1WIs and a fast spin echo sequence system for T2WIs. The cervical coil was used. The slice width was 4 mm, and the acquisition matrix was 512 × 256. The sequence parameters were repetition time 612 ms/echo time (TE) 13 ms for T1WIs and repetition time 2,400 ms/echo time 114 ms for T2WIs. In every case, a preoperative MRI was performed to define the Modic classification, which was classified into group Modic-1 or Modic-2 (Figures 1 and 2). Modic-3 changes were not seen in this series. An additional group without vertebral endplate changes on MRI was designated Modic-0 (Figure 3), which was added to this classification. All of the data were collected and reviewed by two orthopedic surgeons (YL and JW).

### Surgical technique

All patients received ACDF by the same senior surgeon. Surgical procedures were carried out using the anterior approach via a right-sided skin incision. For the purpose of adequate neural decompression, the posterior longitudinal ligament must be excised completely. The endplates were resected with a curette or burr. The polyetheretherketone (PEEK) cage (Medtronic Sofamor Danek, Memphis, TN) or tricortical iliac crest graft was used, which was filled with local bone fragments from the decompression and inserted into the disk space, and the anterior plate system was applied (Medtronic Sofamor Danek, Memphis, TN).

### Evaluation criteria

Clinical data were prospectively collected preoperatively and at 3, 6, 12, and 24 months after surgery. When the follow-up was longer than 2 years, the last data available were used for statistical analysis.

The modified Japanese Orthopaedic Association (JOA) scoring system [13] was used to determine functional status before surgery and at the final follow-up visit. The recovery rate (%) at the final follow-up visit was calculated by using the Hirabayashi method: (postoperative JOA score − preoperative score)/(17 − preoperative score) × 100%. Normal score of JOA present 17.

The visual analog scale (VAS) was used to determine axial symptoms before surgery and at the final follow-up visit.

### Statistical analysis

All data were collected and the software of by SPSS Version 17.0 was used for the statistical evaluation. Cohen’s kappa statistics was used to calculate intra- and interrater reliability [14]. Statistical analysis included descriptives and multivariate repeated measures analysis of variance with Student-Newman-Keuls test for group-to-group comparisons. Comparisons with values of *P* < 0.05 were considered statistically significant. Results were presented as mean ± standard deviation.

## Result

In this study, 106 patients were included who were followed up for a mean period of 30.2 months (range, 24–38 months). On preoperative MR images, 23 (21.7%) patients were classified as Modic-1, 39 (36.8%) patients were classified as Modic-2, and 44 (41.5%) patients were classified as Modic-0 as shown in Table 1. The intraobserver agreement with the Modic classification was excellent (weighted kappa 0.86). The interobserver agreement was substantial (weighted kappa 0.73). There were no cases of intraoperative complications or major neurological or vascular, pseudoarthrosis, or wound complications. No patient needed additional cervical decompression surgery due to recurrent or residual symptoms.

**Table 1:** **Demographic and baseline information**

	**Modic**			**Total**
	**0**	**1**	**2**	
Age (years)	55.3 ± 7.2	53.6 ± 5.7	58.5 ± 6.5	55.8 ± 6.5
Sex				
Men	24	13	20	57
Women	20	10	19	49
Operated level				
C4-C5	11	5	7	23
C5-C6	18	12	21	51
C6-C7	15	6	11	32

No significant difference between groups was found in age, sex, or operated level.

Generally, the preoperative mean JOA score was 9.8 ± 2.6, 9.3 ± 2.1, and 9.0 ± 2.8, respectively. At the final follow-up, the mean JOA score significantly increased to 14.2 ± 2.1, 14.6 ± 1.9, and 14.1 ± 0.9, respectively, representing a statistically significant difference (*P* < 0.05). At the last follow-up, the mean recovery rates were 61.1%, 68.8%, and 63.8%, respectively. Comparing the result of mean JOA score after ACDF in the Modic-1 group and other groups, statistically significant differences could be found at 12 months after surgery (*P* < 0.05). There were no significant differences of JOA score among the three groups before surgery and 3, 6, and 24 months after surgery, which are summarized in Table 2.

**Table 2:** **Evolution of JOA according to Modic changes**

**JOA**	**Time**				
	**Preoperative**	**3 months**	**6 months**	**12 months**	**24 months (min)**
Modic-0	9.8 ± 2.6	11.5 ± 2.7^†^	13.0 ± 3.2^†^	14.5 ± 2.3^†^	14.2 ± 2.1^†^
Modic-1	9.3 ± 2.1	11.8 ± 1.9^†^	13.4 ± 2.1^†^	14.7 ± 1.7^†*^	14.6 ± 1.9^†^
Modic-2	9.0 ± 2.8	11.2 ± 0.7^†^	12.8 ± 0.5^†^	14.3 ± 1.1^†^	14.1 ± 0.9^†^

^†^Significantly different from the preoperative (*P* < 0.05).

*Significantly different from the Modic-0 group (*P* < 0.05).

The preoperative VAS of axial symptoms was 7.7 ± 2.3, 7.6 ± 2.1, and 7.1 ± 1.5, respectively. There was no significant difference in axial symptoms among the three groups before surgery. At the final follow-up, the VAS of axial symptoms significantly decreased to 2.0 ± 1.5, 1.5 ± 1.1, and 2.1 ± 1.6, respectively, representing a statistically significant difference (*P* < 0.05). Comparing the outcome VAS of axial symptoms among different groups after ACDF, patients in the Modic-1 group reported significantly lower VAS of axial symptoms at 3, 6, 12, and 24 months postoperatively (*P* < 0.05), which are summarized in Table 3.

**Table 3:** **Evolution of axial symptoms according to Modic changes**

**VAS**	**Time**				
	**Preoperative**	**3 months**	**6 months**	**12 months**	**24 months (min)**
Modic-0	7.7 ± 2.3	3.0 ± 2.1^†^	2.3 ± 1.8^†^	1.8 ± 1.5^†^	2.0 ± 1.5^†^
Modic-1	7.6 ± 2.1	2.5 ± 1.9^†*^	1.9 ± 1.7^†*^	1.3 ± 1.1^†*^	1.5 ± 1.1^†*^
Modic-2	7.1 ± 1.5	3.5 ± 2.2^†^	2.4 ± 1.9^†^	1.8 ± 1.6^†^	2.1 ± 1.6^†^

^†^Significantly different from the preoperative (*P* < 0.05).

*Significantly different from the Modic-0 group (*P* < 0.05).

## Discussion

The previous studies focused on the relationship between lumbar spine and Modic changes. Only a few articles had reported prevalence of Modic changes in the cervical spine. Peterson et al. [11] reported that Modic changes were seen in 19 of the 118 patients with cervical spine disease (16%). Modic-1 changes were found in 13 patients, which were the most common type. Modic-3 changes were found in five patients, which were the second common type. Modic-2 changes were found in three patients. On the contrary, Mann et al. [15] reported the Modic changes were seen in 172 of 426 patients with cervical spine disease (40.4%). Modic-2 changes were the most common type. Matsumoto et al. [16] conducted a study on asymptomatic subjects, and Modic-2 changes were more common than Modic-1. The age and clinical symptoms were attributed to the differences of results in the abovementioned studies. In our study, Modic-2 changes were found in 39 (36.8%) patients, which were more common than Modic-1 changes. Modic-3 changes were not referred in this study.

Several researchers tried to correlate Modic changes with clinical outcome following lumbar arthroplasty or fusion. There were controversial results between Modic changes and chronic low back pain, especially for Modic-1 changes [17,18]. Esposito et al. [9] reported that patients with Modic changes were treated by anterior interbody fusion. The patients with type 1 changes achieved better result compared to patients with type 2 changes. Similar results were reported by Chataigner et al. [19] and Buttermann et al. [10]. These fusion surgery studies indicated that Modic-1 changes might represent a positive prognostic factor after lumbar surgery. In contrast, the result of Siepe’s study [20] demonstrated that clinical outcomes of patients with Modic changes were not significantly better than others. Whether the clinical outcome of patients with Modic-1 or Modic-2 changes was better than others without Modic changes was still unknown.

To our knowledge, there are many similar properties between the cervical and lumbar spine, such as morphology, activity, and the lordosis of physiological curvature, which were spinal degeneration predilection sites. The Modic changes of the cervical spine are similar to those of the lumbar spine. Degenerative marrow changes on MRI also have an obvious correlation with degenerative disk disease. Compared with the lumbar spine, the cervical spine has higher degree of global and intersegmental activities. It was reported that the damage of cartilage endplates and bone marrow in the cervical spine was caused by torsional forces. Modic changes were influenced by the structural deterioration of the intervertebral disks caused by mechanical stress. According to the Modic changes, the most common level was the C5-C6, which was the most flexible level [11,15,16]. Therefore, hyperactivity may be the main reason for cervical Modic changes.

In this study, all patients received ACDF surgery, and the patients with Modic-1 changes group had better outcomes, especially in axial symptoms. During the operation, the majority of the inflammatory disk tissue was resected in the patients with Modic-1 changes, which is more effective in axial symptom relief in comparison to the patients with Modic-2 changes. Anterior interbody fusion with graft had reconstructed the stability of cervical spine, inhibiting further damage to the cartilage endplates. In addition, natural evolution of degenerative endplate disease may cause differences in axial symptom relief between Modic groups. The patients in the Modic-1 group had a shorter disease duration compared to that of patients in the Modic-2 group.

### Limitation

This study was a retrospective study with a small sample size. The prospective and large-scale studies should be performed to confirm the result. In the future study, we can explore the correlation between Modic changes and curvature on the cervical spine.

## Conclusion

According to our study, ACDF provides satisfactory results in the treatment of CSM. There was significant clinical improvement regardless of preoperative Modic type. When we analyzed outcomes based on different Modic types, the best clinical outcome in patients with Modic-1 changes was better than that in patients with Modic-2 and Modic-0 changes.
